# An Aneurism of the Aorta, *with Cursory Remarks on Various Morbid Affections of the Heart*

**Published:** 1804-04-01

**Authors:** John Moodie


### An Aneurism of the Aorta, *with cursory Remarks on various morbid Affections of the Heart*; *by* John Moodie, M. D. &c.

JOHN SMITH, aged thirty-four, of a healthy constitution, by trade a carpenter, serving with a detachment of the Royal Regiment of Artillery, in the East Indies.

On the 15th of November, 1783, he was sent into the Hospital at Mangalore, having a large aneurism of the aorta, apparently making its way though the sternum, and raising up part of the right pectoral muscle to the magnitude of a large fist.

The tumour was attended with a strong pulsation, and had been gradually increasing from its first appearance. About three years previous to this time, as appeared from the account given by the patient himself, he had received a kick from a horse on the left breast, to which may probably be ascribed the cause of the aneurism. And it doubtless increased more rapidly than it otherwise would have done by great exertion, and the pressure of heavy and hard bodies against the chest, to which his business subjected him; and seems to have accelerated the alarming symptoms which now threatened the life of the patient.

In order to lessen the momentum of the circulation, and prevent as much as possible the disorder from increasing, repeated bleeding, gentle laxatives, with a spare diet, was directed, and he was cautioned to refrain from the use of spirituous liquors, and every kind of bodily exertion, or mental agitation. But notwithstanding every attention which could be paid to his case, when the tumour was examined on the 30th, it was found that the skin of the apex was broke, so that a fatal hæmorrhage was hourly expected.

However, his appetite being good, his rest undisturbed, and in other respects healthy, though warned of the event, so far was he from a sense of danger, that he thought the Medical Gentlemen had mistaken his case, saying, "he believed he should cheat them all." In this situation he continued till the 4th of December, when the blood suddenly discharged itself from the tumour in a considerable quantity, and with great impetuosity, but the hæmorrhage was for the present restrained by the application of dry lint introduced pretty tight into the orifice, and retained by the gentle pressure of a bandage.

 Nevertheless,

Nevertheless, this alarming circumstance did not prevent him from eating freely of animal food at supper; about two hours afterwards, being perfectly easy, he went to bed. Here it may be supposed, that in a short time the impetus of the blood might be increased by the addition of new chyle, which in how small a degree soever, yet sufficient to overcome the resistance of the pressure made upon the part. Accordingly, in little more than half an hour, a quantity of concreted mass was forced from the orifice, and the blood gushed out in a full stream, when the patient suddenly turned upon his side, and instantly expired.

Upon opening the cavity of the thorax, the heart was found in its natural state, excepting some stoney concretions which appeared to have been recently formed. The lungs, though paler than usual, had no marks of disease; but on moving them aside, so as to bring the aorta into view, we observed that the cyst of the tumour was evidently formed by a dilatation of the aorta at its egress from the heart, but in a greater degree at its curvature, and from thence continued along the *aorta decendens* to the vertebræ of the back, where, all at once, it assumed the natural appearance. The carotid and subclavian arteries were a little ossified.

The aneurismal sac was ossified in several places, and by its continual pressure upon the vessels embracing the periosteum of the spine and sternum, had rendered both those parts carious, and entirely made its way through the latter. And it can hardly be imagined that the arterial coats were capable of a distension so great as to reach quite through the sternum to the surface. However I have reason to think, that the last mentioned circumstance was occasioned by the blood having gradually escaped from the artery, and by its force condensed the cellular membrane under the sternum, so as to form it into a cyst.

An aneurism, or a preternatural enlargement of the heart, or its cavities, is a disease of frequent occurrence. But although the subject is extremely interesting in the practice of medicine, it is seldom much remarked or well understood. Hence it is of the utmost importance to be able to distinguish an internal aneurism from various other affections which it so nearly resembles; for after attentively considering all the symptoms of this deplorable disorder, we must in general acknowledge our inability to discover any appearances sufficiently unequivocal for this purpose.

Such

Such information therefore can only be derived from a comparison of a variety of facts illustrative of the subject.

While the force of the heart exceeds the resistance of the arteries, it continues of the same dimensions; but when the resistance of the latter, from becoming rigid and bony, exceeds the force of the heart, its cavities then enlarge. Hence practical anatomy furnishes us with many observations, shewing that the heart is thus frequently distended: and we find one in the Philosophical Transactions, where the left ventricle was observed to be three times larger than the right. Fernelius (in his Pathologia, lib. v. chap. 12) relates an uncommon case of this kind; where he says, that the frequent concussions of the heart were so violent and strong as not only to luxate, but even to break some of the adjoining ribs.

Marchettis, in his Anatomy, informs us, that he found a heart so large as to possess the whole thorax, and the ventricles of such a prodigious extent, especially the right, that a natural sized heart might be contained in it. Of the causes of this preternatural distension of its ventricles, one may be the air, which is not unfrequently found in the cavities of the heart, enlarging them immensely.

In a woman who died suddenly, Ruysch found the heart of vast magnitude, from the air with which it was distended, containing scarcely any blood, as appeared from inserting the point of a knife into it, the heart suddenly subsiding, as if a bladder full of air had been punctured.— Dissections convince us, that in the heart have been found inflammation, suppurations, erosions, concretions, and various other morbid affections, which may exist for a considerable time without any alarming symptoms. From the Academ. des Sciences, we have an instance of this kind, where, upon opening the body of the Duke of Brunswick, the heart was found eroded by ulcers, and the right ventricle appeared burst, from such an ulceration and erosion. To the above may be added the death of our late King. When the cavity of the thorax was opened and inspected by the serjeant surgeons, they found the right ventricle of the heart actually ruptured, and a considerable quantity of blood discharged through the aperture into the pericardium, so that he must have died instantaneously, in consequence of the effusion.

This appears the more remarkable, as it happened to a prince of a healthy constitution, unaccustomed to excess, and far advanced beyond that period of life when the

 blood blood might be supposed to flow with a dangerous impetuosity.

Morand, in the Academ. des Sciences above mentioned, relates a very uncommon case. When investigating the cause of sudden death in the body of a gentleman, there appeared, upon opening the pericardium, a large quantity of coagulated blood, and in the left ventricle a perforation, which was equal to eight lines in length, and the fleshy substance of the heart appeared so tender, that the probe made its way through in every part by the slightest pressure.

Upon examining the histories of this disease, we find, that aneurisms have generally arisen from blows, contusions, and other injuries about the chest, especially from overstretching the coats of the arteries by violent straining, or from erosion; instances of which may be seen in Lancisius. The celebrated Morgagni has also related some interesting cases of this disease; but more would be superfluous on the present subject.

When pointing externally, surgeons should be extremely careful in distinguishing this kind of tumour from others of a less dangerous nature; since it is known, that fatal instances have happened by opening them: an instance of which, I myself saw many years ago, where the patient expired a few hours after the operation. And since my residence in this city, I was consulted by a lady who had an external aneurism situated above the spine of the ilium, which appeared sufficiently capacious to contain upwards of three pints, and was doubtless filled with blood. Although I could not have supposed it possible that the nature of this tumour could have been mistaken by a medical man, nevertheless I was informed that a surgeon had proposed to open it.

I only advised bleeding, a low diet, and rest, with a compress wet in a solution of salt in water, applied to the part three times a day, and secured by the gentle pressure of a bandage. By means of the above, in a short time, the tumour entirely disappeared. That such tumour is an aneurism, may certainly be known from its being seated in a part where we know, from the anatomical structure of the body, that there is some large artery near, but especially when it has a manifest pulsation, also if the tumour be of the natural colour of the skin, diminishes by slight pressure, and returns again when that is removed.

But an aneurism of the internal parts of the body is inaccessible; all that can be done for the patient, is to prevent, vent, as much as possible, the disorder from increasing by the treatment in the aforementioned case.

When the cause of a gangrene in the extremities of old people is occasioned by a debility of the functions of the heart, so as to be unable to propel the blood into the extremities, we may despair of any effectual relief.

From polypous concretions, whether formed in the heart or its larger vessels, arise many irregular and painful symptoms; and there is, unfortunately, no cure suggested by authors for this dreadful disorder.

*Bath, Feb.* 20, 1804.

